# Data driven theory for knowledge discovery in the exact sciences with applications to thermonuclear fusion

**DOI:** 10.1038/s41598-020-76826-4

**Published:** 2020-11-16

**Authors:** A. Murari, E. Peluso, M. Lungaroni, P. Gaudio, J. Vega, M. Gelfusa

**Affiliations:** 1 Consorzio RFX (CNR, ENEA, INFN, Università di Padova, Acciaierie Venete SpA), Corso Stati Uniti 4, 35127 Padua, Italy; 2 Department of Industrial Engineering, University of Rome “Tor Vergata”, via del Politecnico 1, 00133 Rome, Italy; 3 Laboratorio Nacional de Fusión, CIEMAT, Av. Complutense 40, 28040 Madrid, Spain

**Keywords:** Magnetically confined plasmas, Experimental nuclear physics, Characterization and analytical techniques, Information theory and computation

## Abstract

In recent years, the techniques of the exact sciences have been applied to the analysis of increasingly complex and non-linear systems. The related uncertainties and the large amounts of data available have progressively shown the limits of the traditional hypothesis driven methods, based on first principle theories. Therefore, a new approach of data driven theory formulation has been developed. It is based on the manipulation of symbols with genetic computing and it is meant to complement traditional procedures, by exploring large datasets to find the most suitable mathematical models to interpret them. The paper reports on the vast amounts of numerical tests that have shown the potential of the new techniques to provide very useful insights in various studies, ranging from the formulation of scaling laws to the original identification of the most appropriate dimensionless variables to investigate a given system. The application to some of the most complex experiments in physics, in particular thermonuclear plasmas, has proved the capability of the methodology to address real problems, even highly nonlinear and practically important ones such as catastrophic instabilities. The proposed tools are therefore being increasingly used in various fields of science and they constitute a very good set of techniques to bridge the gap between experiments, traditional data analysis and theory formulation.

## Introduction

After a brief period, mainly dominated by observational methods, the scientific enterprise has progressed in a hypothesis driven way, particularly in fields such as physics and chemistry. Typically, on the basis of already established theories, new models have been developed mathematically and their predictions have been falsified with specifically designed experiments^1,2^. This scientific methodology has been very successful and has produced great results, particularly in the investigation of mostly deterministic linear systems, composed of weakly coupled parts. On the other hand, in the last decades, various technological and cultural changes have contributed to expose the limitations of such an approach to knowledge discovery. First, all scientific disciplines are nowadays required to tackle increasingly challenging, nonlinear problems and systems, some of which, like thermonuclear plasmas, are very difficult, if not impossible, to model with theories based on first principles. Moreover, many newly interesting phenomena, for various reasons ranging from intrinsic randomness to inaccessibility for measurement, are characterised by a high level of uncertainties, limiting the effectiveness of purely deterministic approaches. All this in the context of a data deluge, affecting not only society in general but also the scientific community^3,4^. Increasingly many experiments, particularly in Big Physics, tend to produce enormous amounts of data, impossible to properly analyse by hand or with traditional statistical tools, conceived in an era of paucity of information. At CERN, the main detector ATLAS has shown the capability of producing Petabytes of data per year. In its prime, the Hubble space telescope sent to earth Gigabytes of data per day and the data warehouse of the Joint European Torus (JET), the largest operational Tokamak in the world, is approaching 0.5 Petabytes.

The limitations of the hypothesis driven approach to investigating complex physical objects, affected by great uncertainties, has become particularly evident in the case of open systems such as high temperature plasmas^5,6^. Thermonuclear plasmas are open, nonlinear, out of equilibrium systems characterised by a very high level of complexity and poor accessibility for measurement in a hostile environment. The consequences are typically a limited experimental characterization of many phenomena and the presence of significant noise in the data. Such complexity and high levels of uncertainty in practice reduce dramatically the effectiveness of formulating theories from first principles. Traditional analysis techniques, such a simple fitting routines or log regression, are also of limited help, due to their rigidity and poor exploratory capability. They indeed assume that a solution of the problem is already known and that only the parameters of the models have to be adjusted. As a consequence, a lot of untapped knowledge remains buried in the large collected databases, with the scientists unable to fully exploit them due to the lack of adequate mathematical tools for data mining. Historically, these difficulties have led to a hierarchy of descriptions (particle, kinetic, fluid) and to a plethora of ad hoc models, aimed at interpreting specific phenomena with poor generalization power and limited applicability^6^. On the other hand, modern machine learning techniques, as deployed in commercial applications, are not completely adequate to address scientific questions. This quite unsatisfactory situation motivates the quest for more sophisticated analysis methodologies to draw reliable and sound conclusions.

With regard to the rest of the paper, in the next section the present limitations of traditional machine learning (ML) methods are discussed. In “Data driven theory with symbolic regression via genetic programming” section, the main tools developed in the last decade, to overcome the drawbacks of commercial data mining techniques, are described in some detail. Numerical examples, to show the potential of the new approaches for scientific investigations, are the subject of “Numerical examples” section. In “Application to scaling laws: the energy confinement time in Tokamaks” and “Application to the extraction of boundary equations: disruptions” sections, two fundamental applications to Magnetic Confinement Nuclear Fusion are presented: the scaling laws for the energy confinement time and an original derivation of the boundary equation between safe and disruptive regions of the operational space. Conclusions and lines of future investigations are the subject of the last section of the paper.

## The limits of traditional statistical and machine learning tools

In the last years, the limitations of the hypothesis driven approach to scientific investigation has motivated the adoption of more data drives techniques. Typically referred to with the collective name of “machine learning”, these methods are explicitly conceived to derive models directly from the data^7,8^. The developed tools make a different use of the available computational power. Whereas traditional algorithms implement complete solutions to the problem at hand and mainly help only in carrying out massive calculations, machine learning tools are exploratory in nature, in the sense that they analyse the data to find possible, not already known solutions and models. In the last years, their successes have been astonishing and they are now widely deployed in a great variety of domains, ranging from automatic translators to image and voice recognition, to diagnostic support. On the other hand, even if machine learning tools have found many very useful applications, they are popular in the private sector but their penetration in the sciences has been quite poor and typically confined to “theory-less” applications, where it is not required to devise interpretable mathematical models. This relatively minor acceptance is the consequence of the many limitations of traditional machine learning techniques. Indeed, to be really useful, the knowledge discovery process in the natural sciences has to satisfy specific criteria and requirements, which are not necessarily crucial in other fields. In particular, in addition to the accuracy of prediction, the derived equations must reflect the “physics” reality of the phenomena under investigation. The derived models should also be easily interpretable and guarantee a proper treatment of the uncertainties and a solid quantification of the confidence intervals. Even if the traditional data driven tools are providing quite impressive performance in terms of prediction accuracy, they have been found lacking in practically all the other respects. The main problem relates to the structure of their mathematical models, which can be completely unrelated to the physics reality of the investigated phenomena. This poor “physics fidelity” is a major concern, which has badly affected the adoption of many machine-learning tools in various scientific disciplines, particularly in physics and chemistry. Indeed, a significant dissonance, between the model mathematical form and the actual physics of the phenomena under study, can compromise some of the most important objectives of scientific investigations. Indeed, it can jeopardize the interpretation of the results in the light of existing mathematical theories, with consequent reduced contribution to general understanding and limited confidence in the extrapolability of the results. This aspect is particularly problematic for the design of new experiments, which are typically required to investigate the phenomena in previously unexplored regions of the operational space. Purely statistical models, without any relation with the actual dynamics of the systems under study, can be delicate to use in this perspective and can provide misleading indications.

## Data driven theory with symbolic regression via genetic programming

To overcome, or at least to alleviate, all the previously mentioned limitations of machine learning for science, a new methodology has been developed in the last decade. It is called Symbolic Regression (SR) via Genetic Programming (GP)^9^. This technique consists of a series of tools, which allow a new approach to theory formulation. The mathematical models, describing the phenomena under investigation, are derived directly from the data. The tools can be used either in an exploratory way, by reducing to a minimum the “a priori” assumptions, or steered toward certain classes of solutions, for example for comparison with existing theories. They implement genetic programming but apply it to the symbolic manipulation of mathematical formulas. Genetic Programs (GPs) are designed to emulate the behaviour of living organisms by working with a population of individuals, i.e. mathematical expressions in our case^10,11^ The individuals are candidate models of the system to be investigated. These models are represented as trees, which makes easy the implementation of Genetic Programming, in particular of the three main genetic operators: copy, cross-over and mutation. The main mechanism of the methodology consists of traversing the database and checking the behaviour of each candidate model, derived from the initial families of functions selected by the scientists. These initial families, i.e.basic units, are usually arithmetic operations, functions, possibly including saturation terms, and ad hoc operators introduced by the user. The basis functions have to be selected carefully, taking into account the nature of the problem at hand. For example, their combination must include a realistic and physically meaningful model of the phenomena to be analysed. For the sake of completeness, the basis functions implemented to obtain the results presented in the rest of the paper are summarized in Table 1.

**Table 1:** Basis functions manipulated with symbolic regression via genetic programming to obtain the mathematical models presented in this paper.

Function and operator class	List
Arithmetic operators	constants, + ,−,*,/
Exponential functions	exp(x_i_),log(x_i_),power(x_i_, x_j_), power(x_i_,c)
Squashing functions	logistic(x_i_),step(x_i_),sign(x_i_),gauss(x_i_),tanh(x_i_), erf(x_i_),erfc(x_i_)

**Figure 1:**
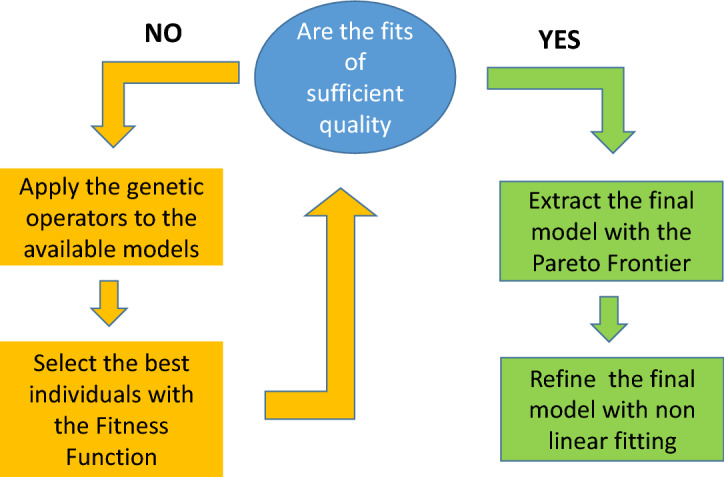
Overview of the proposed methodology to identify the best models, directly from the data. Figure created using Microsoft Power Point from Office Professional Plus 2013 https://www.microsoft.com/it-it/microsoft-365/microsoft-office?rtc=1.

A specific metric must be used to evaluate the performances of the candidate equations. This performance indicator, designed to find the appropriate trade-off between complexity and goodness of fit, is usually called fitness function (FF) and allows selecting the best candidates of each generation. The better the FF of certain individuals, the higher the probability to choose those elements to produce descendants. The genetic operators are applied to these best performing individuals to obtain the following population. The process is iterated for a high number of generations, until convergence on a satisfactory solution; Fig. 1 reports graphically what has been described in the above paragraphs.

In the end, the proposed technique provides a series of data driven models, whose mathematical structure is the most appropriate to fit the available databases. On the other hand, “a priori” knowledge can be brought to bear on the formulation of the final solutions. Indeed, the extraction of the models can be influenced at least at three different levels: (a) the selection of the basis functions (b) the structure of the trees (c) the mathematical from of the fitness function.

Of course, the heart of the method is the FF, the indicator chosen to assess the quality of the solutions. To this end, various model selection criteria have been implemented: the Akaike Information Criterion (AIC), the Takeuchi Information Criterion (TIC) and the Bayesian Information Criterion (BIC)^12^. Since all these metrics are conceptually similar, only the AIC is discussed in the following. The aim of the AIC estimator is to minimize the generalisation error by finding the best trade-off between complexity and goodness of fit. The most widely used form is:

1 $$ AIC = n \cdot {\text{ln}}\left( {MSE} \right) + 2k $$

where the Mean Square Error (*MSE*) is evaluated between the predictions obtained using each model and the target data, while *k* represents the complexity of the model itself, in terms of number of nodes of the tree, and *n* stands for the number of columns or rows, i.e.entries, in the database to be analysed. Considering the parameterization of Eq. (1), it can be easily understood why this indicator has to minimized. The closer the models are to the data (first addend) and the lower the number of nodes is required to express them in a tree form (second addend), the lower the estimator. Therefore parsimony is built in the metric to avoid overfitting, contrary to the vast majority of the alternatives (for example the maximum likelihood), which do not consider this issue and therefore do not include terms penalising excessive complexity of the models.

In terms of computational complexity, it should be considered that the proposed genetic programming approach can be parallelized relatively easily. Therefore, the main difficulties, in the deployment of symbolic regression, typically reside in the quality and quantity of the data. The databases available are often not sufficiently selective to identify a unique model, clearly superior to all the others in all respects. The most common situation is convergence on a small set of reasonable candidates. The typical approach to select the final model is based on the so-called Pareto Frontier (PF)^13^, which consists of the set of best models, each one for a specific value of complexity. When the FF of the models in the PF is plotted versus complexity, the resulting curve typically allows identifying a region with the best trade-off between goodness of fit and complexity; the models in this region are the ones, to which a final nonlinear fitting^14^ is applied to determine the most appropriate solution. This last stage allows also quantifying the confidence intervals to be associated to the models. More details on the entire procedure can be found in the literature^15,16^.

## Numerical examples

To illustrate the potential of the proposed techniques, in this section some numerical examples are analysed, using synthetic data. It is worth mentioning that the numerical tests reported have been performed in conditions very similar to those of the real life cases, presented in the rest of the paper. The number of generated examples is of the order of a couple of thousand. Zero mean Gaussian noise of standard deviations equal to about 10% of the data mean value has been added, again to simulate realistic experimental situations.

The first case involves the identification of scaling laws, mathematical expressions meant to quantify how the properties of a system change with size. Their importance is at least twofold. On the one hand, scaling laws reveal important aspects about the behaviour and dynamics of the systems under investigation. From a more practical perspective, scaling considerations play a fundamental role in the engineering of artefacts and machines. Extracting robust scaling laws directly from available data is essential in the case of the design of new experiments, which cannot be easily modelled theoretically, such as Tokamak devices. Unfortunately, the mathematical methods, available until the development of SR via GP, were basically fitting algorithms, which required the scientist to make strict assumptions about the mathematical form of the scalings. Consequently, for decades the most popular forms of the scaling laws have been power laws in the regressors, mainly because a popular numerical tool, log regression, was available to derive them^17^. Until recently, indeed, all scaling laws in Tokamak physics were basically power laws^18^. Even if very popular, power laws present various drawbacks of high significance for scientific applications. First of all, tens of different physical mechanisms end up providing scalings in power law form. Therefore, power laws are poorly informative about the actual dynamics behind the scalings^19^. Moreover, power laws do not present any saturation mechanism, are monotonic in the regressors (cannot model minima or maxima) and force the interaction between the independent variables to be multiplicative. This application of SR via GP is therefore a paradigmatic example of the much higher flexibility provided by the new developed solutions, compared to previous techniques. To show the potential of the developed tools, a series of systematic tests has been performed, proving the capability of the methodology to derive scaling laws of any mathematical form provided enough data is available. Various functional relations have been implemented for generating synthetic data, to assess how the proposed technique manages to identify the right equation. As a representative example, the results obtained for a model consisting of three parts is discussed in the following. Equation (2) contains a power law term, a linear term and a squashing factor, covering some of the most important functional dependencies of practical relevance in the sciences. The mathematical expression of this hypothetical scaling is:

2 $$ {\text{f}}\left( {{\text{x}}_{1,2,3,4,5,6} } \right) = \frac{{{\text{x}}_{1}^{3.5} {\text{x}}_{2} }}{{{\text{x}}_{3}^{1.5} }} + {\text{x}}_{4} - 5 \cdot {\text{x}}_{5} + \frac{10}{{1 + {\text{e}}^{{ - 0.15 \cdot {\text{x}}_{6} }} }} $$

It should be mentioned that Eq. (2) is at least of the same level of complexity as the actual experimental cases analysed in the rest of the paper. The method has been able to easily recover the right expression of the scaling in very reasonable time, whereas log regression is obviously at a loss to identify this type of function.

To complement the previous example, a series of additional tests have been performed to verify that the developed tools can identify the most appropriate dimensionless quantities to describe a database. Dimensionless quantities are quantities of dimension 1, which are often utilized to simplify the description of complex systems, characterized by multiple interacting phenomena. Their importance is emphasized by the Buckingham π theorem, which states that the validity of the laws of physics does not depend on a specific unit system. Therefore, any physically meaningful equation can always be expressed as an identity involving only dimensionless combinations, obtained by extracting ratios and/or products of the variables linked by the law. Dimensionless quantities have proved to be particularly useful in fluid dynamic. A well-known law connecting dimensionless quantities is:

3 $$ Pe = Pr \cdot Re $$

The Peclet number *Pe* is used to evaluate the ratio between transferred heat by advection and diffusion in a fluid. The Prandtl number *Pr* is defined as the ratio of kinematic and thermal diffusivity; the Reynold number *Re* takes into account the relative importance of viscosity for the internal layers of a fluid. The above quantities can be written as:

4 $$ Re = \frac{\rho \cdot u \cdot d}{\mu } $$

5 $$ Pr = \frac{{\mu \cdot c_{p} }}{k} $$

where:

1. μ is the dynamic viscosity
2. k is the thermal conductivity
3. $c_{p}$ is the specific heat
4. ρ is the density
5. u is the velocity of the fluid
6. d is a characteristic linear dimension of the object in which the fluid moves

To prove the capability of the proposed methodology to identify dimensionless variables, a set of data has been generated using Eq. (3). On the other hand, SR via GP has been provided only with the dimensional quantities, among which of course the ones used to build the database, i.e. the dimensional quantities appearing in Eqs. (4) and (5). The implemented algorithm has been able to derive the dimensionless relationship (3) by grouping the correct dimensional variables for various levels of normally distributed noise. As an example, the following scaling has been obtained with 30% of added noise:

6 $$ Pe = 0.99 \cdot \left( {\frac{\rho \cdot u \cdot d}{\mu }} \right)\left( {\mu \frac{{c_{p} }}{k}} \right) $$

which, once rounded out, provides the exact answer, Eq. (3).

## Application to scaling laws: the energy confinement time in Tokamaks

Nuclear fusion, the process of building larger nuclei from the synthesis of smaller ones, is considered a potential solution to humanity energy needs^20^. The most promising technical alternative, to bring the nuclei close enough to fuse, is magnetic confinement. In this approach, plasmas are confined by magnetic fields in a vacuum chamber and heated to temperatures higher than in the core of the sun. The best performing configuration of the magnetic fields so far is the Tokamak^5^. One of the most crucial quantities, to assess the reactor relevance of a magnetic configuration, is the so called energy confinement time τ_E_, which quantifies how fast the internal energy of the plasma is lost^21–23^. Unfortunately, high temperature plasmas are too complex to be modelled, even numerically, to estimate τ_E_. The range of scales involved spans many orders of magnitudes, ranging from microturbulence to macroscopic dimensions comparable to the size of the devices. For this reasons, from the beginning of the 70′s, scientists have spent many efforts trying to derive the behaviour of τ_E_ from experimental data using robust scaling laws, since τ_E_ is calculated routinely in practically all Tokamaks. Therefore, multi-machine databases, with reliable estimates of this quantity, are available. Moreover, to reduce the adverse effects of the uncertainties in the measurements, very significant efforts have been devoted to formulating the scaling laws of τ_E_ in terms of dimensionless variables, which are believed: (a) to have a stronger physical base with respect to dimensional scalings, and (b) to be more robust, particularly for extrapolation. The most commonly used dimensionlees quantities have been derived from the so called Vlasov equation. This model assumes that the plasma behaviour is governed by equations invariant under a certain class of transformations. Consequently, also any scaling expressions should present the same invariances. Assuming the validity of the assumptions previously cited, the choice of specific dimensionless variables is determined a priori from theoretical considerations and not derived from the data^24,25^.

Recently SR has been applied to the task of identifying potential scaling expressions of the confinement time studying the ITPA DB3v13f^26^ international database, which has been built with the purpose of supporting advanced studies and includes validated measurements from all of the most relevant reactors in the world. In line with what stated above and with the literature^27^, the following dimensionless quantities have been considered:$\beta ,\varrho ,\nu ,,\kappa_{a} ,M,q_{95}$. In the previous list, $\beta$ is the normalized plasma pressure, ρ indicates the normalized ion Larmour radius, ν the normalized collision frequency, $\varepsilon$ the inverse aspect ratio, $k_{a}$ the volume elongation measurement, *M* the effective atomic mass in a.m.u, *q* the plasma safety factor evaluated at the flux surface enclosing the 95% of the poloidal flux^25^. Coherently, the dimensionless product of the ion cyclotron frequency times the confinement time (τ_E_ ω_ci_ ) has been selected as the dependent quantity to be analysed. Using the same selection criteria as in^27^, from which the reference scalings in power law form were derived, the final database is made of 2806 entries.

Table 2 reports the classical scaling expressions^27^ and the one obtained with the aforementioned approach, using SR via GP. The best model, derived with symbolic regression, is not in power law monomial form. The non-power law scaling outperforms the other two according to all the aforementioned estimators (TIC, AIC, BIC, MSE and Kullback–Leibler divergence), normally used to assess the quality of models and fits. The selection process, which converges on the best unconstrained empirical model reported in Table 2 on the basis of the Pareto Frontier, should also guarantee that the risk of overfitting is negligible Since, as mentioned, one of the main purposes of scaling laws consists of providing guidance to the design of new experiments, the predictions for ITER^28^, the next generation international Tokamak at present being built in France, have been investigated in detail. Considering the values in Table 3 for the predicted confinement time on ITER, it emerges how, according to AdNPL scaling, τ_E_ would be about 20% lower than the expected values obtained using the most widely accepted traditional scaling, AdPL1^27^. In terms of simple extrapolations, the model derived with SR via GP is much more in line with the AdPL2 scaling, calculated with the more realistic errors in variable technique^27^. The AdNPL estimate of Table 2 is also confirmed by dimensional scaling laws in terms of dimensional quantities^18^. With regard to interpretation, the non-power law scaling provides a better fit, because the smaller devices follow a more positive trend than the larger ones and the power laws are not flexible enough to accommodate this fact^18,25^.

**Table 2:** Power laws and non power law models in terms of dimensionless quantities. AdPL1 and AdPL2 are two scalings derived from the widely used scaling IPB98(y,2)^27^. The non-power law scaling AdNPL is the one found with SR via GP.

AdNPL	$\tau_{E} \cdot \omega_{ci} = \left( {1.13} \right)_{1.11}^{1.15} \cdot 10^{ - 6} \cdot \frac{{\kappa_{a}^{{1.93_{1.70}^{2.12} }} \beta^{{0.37_{0.33}^{0.41} }} M^{{0.57_{0.46}^{0.67} }} }}{{\rho^{{2.19_{2.16}^{2.22} }} \nu^{{0.40_{0.39}^{0.42} }} q^{{0.16_{0.08}^{0.23} }} }} +$ $- 0.072_{ - 0.085}^{ - 0.060} \cdot \kappa_{a}^{{1.18_{0.94}^{1.40} }} - 0.009_{ - 0.011}^{ - 0.006} \cdot q^{{1.08_{0.94}^{1.21} }} + 0.15_{0.13}^{0.17} \cdot M^{{0.07_{ - 0.05}^{0.19} }}$
AdPL1	$\tau_{E} \cdot \omega_{ci} = 7.214 \cdot 10^{ - 8} \cdot \frac{{M^{0.96}\epsilon^{0.73} \kappa_{a}^{3.3} }}{{\rho^{2.70} \beta^{0.90} \nu^{0.01} q^{3.0} }}$
AdPL2	$\tau_{E} \cdot \omega_{ci} = 1.836 \cdot 10^{ - 8} \cdot \frac{{M^{0.49} \kappa_{a}^{3.34} }}{{\rho^{2.67} \beta^{0.57} \nu^{0.14} q^{1.84}\epsilon^{0.19} }}$

**Table 3:** Comparison of the extrapolated confinement times to ITER. The more flexible model obtained with SR via GP predicts a statistically significant lower ITER energy confinement time than the traditional power laws.

Equation	$ITER\, expected\, \tau_{E} \left[ {\text{s}} \right]$
AdNPL	$2.97_{2.78}^{3.16}$
AdPL1	$3.66$
AdPL2	$3.29$

**Figure 2:**
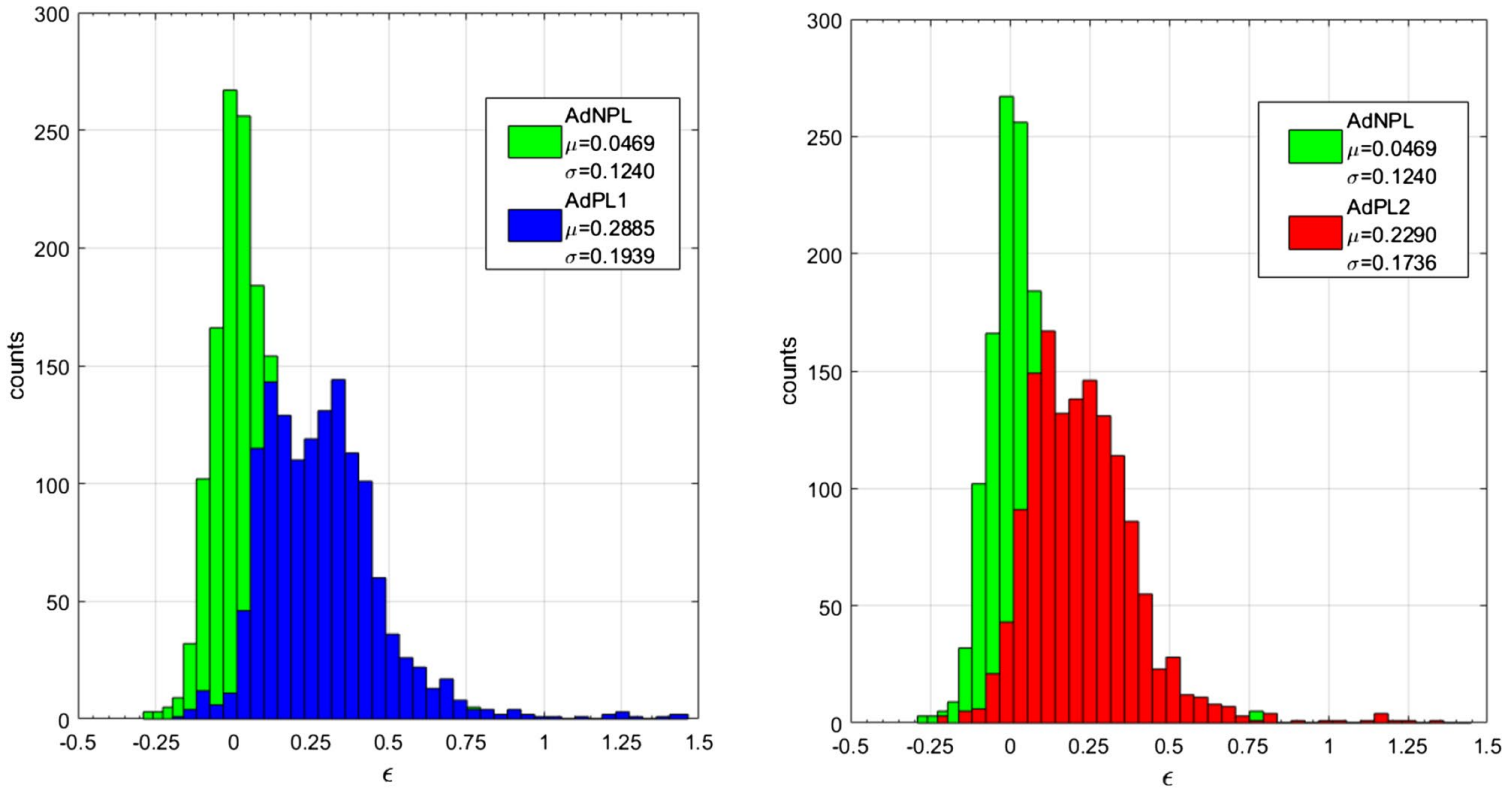
Distribution of residuals between the data of JET and the models predictions. In green, the distribution of the residuals for the non-power law model AdNPL is shown. In blue and red the distributions of the residuals for the models AdPL1 and AdPL2, reported in the literature. Figure created using MATLAB R2019a, https://it.mathworks.com/products/matlab.html.

However, extrapolation is always a delicate matter. The present case is particularly challenging because the parameter space is 7-dimensional and the number of entries less than 3000. The available experimental points are very sparse and this is a well-known difficulty^29^. Therefore, a specific test has been performed. The methodology has been applied on a subset of data (train dataset), without JET’s entries, and then the obtained model has been tested on the previously excluded JET data (test dataset). The traditional scaling laws have been fitted directly to the training dataset and evaluated on the test dataset of JET. The logic behind such an approach resides in the fact that JET parameters cover a middle ground between the smaller devices and ITER. Figure 2 shows the quality of the proposed non power law scaling. The residuals of the AdNPL are better centred on zero and have smaller standard deviation. Extrapolating better, from the smaller devices to JET, gives confidence in the overall quality of the results^30–32^.

## Application to the extraction of boundary equations: disruptions

Many natural and man-made systems can give the superficial impression of being very stable and resilient to perturbations, however, in reality, might be prone to collapse. The consequent catastrophic events may be quite straightforward to interpret and most do not require sophisticated investigations because, adopting the proper level of precautionary care, they are relatively easy to avoid. On the other hand, some such as earthquakes, and in general accidents due to atmospheric phenomena, can be very sudden and very difficult to forecast. In the last years, increasing attention has been devoted to devising mathematical tools more appropriate to investigating and predicting rare catastrophic events^33^. Machine learning tools are new instruments in the arsenal of techniques, which can be deployed by the analysts. The methodology described in the following is proving to be very useful in the modelling and interpretation of sudden disruptive events.

In Magnetic Confinement Nuclear Fusion, the macroscopic instabilities called disruptions are the most striking example of catastrophic failures difficult to predict^5^. They occur when the plasma crosses one of the major stability limits and cause the sudden loss of confinement and the consequent abrupt extinction of the plasma current. They can have very serious consequences for the integrity of the experimental devices; moreover the electromagnetic forces and the thermal loads that they can generate become more severe the larger the machines. Therefore, disruptions are one of the most severe problems to be faced by the Tokamak magnetic configuration on the route to designing and operating commercial reactors. Unfortunately, physical models of disruptions, based on first principles, are practically unusable for prediction. Consequently, in the last decades, machine learning tools have been increasingly deployed to derive empirical models capable of predicting the approach of a disruption^34–40^. Unfortunately, these models present all the problems mentioned in the second section and are particularly lacking in physics fidelity. Indeed, the models of most ML tools have nothing to do with the actual dynamics behind disruptions, even if they can be very accurate in terms of predictions^41–44^. Consequently, the capability of the present models to extrapolate to larger devices is questionable. A method has therefore been developed to combine the predictive capability of the ML tools with the advantages of more physically meaningful equations. The main steps of such an approach are:

1. Training the machine learning tools for classification, i.e. to discriminate between disruptive and non-disruptive examples
2. Determining a sufficient number of points on the boundary between safe and disruptive regions of the operational space provided by the machine learning tools
3. Deploying Symbolic Regression via Genetic Programming to express the equation of the boundary in a physically meaningful form, using the points identified in the previous step.

**Figure 3:**
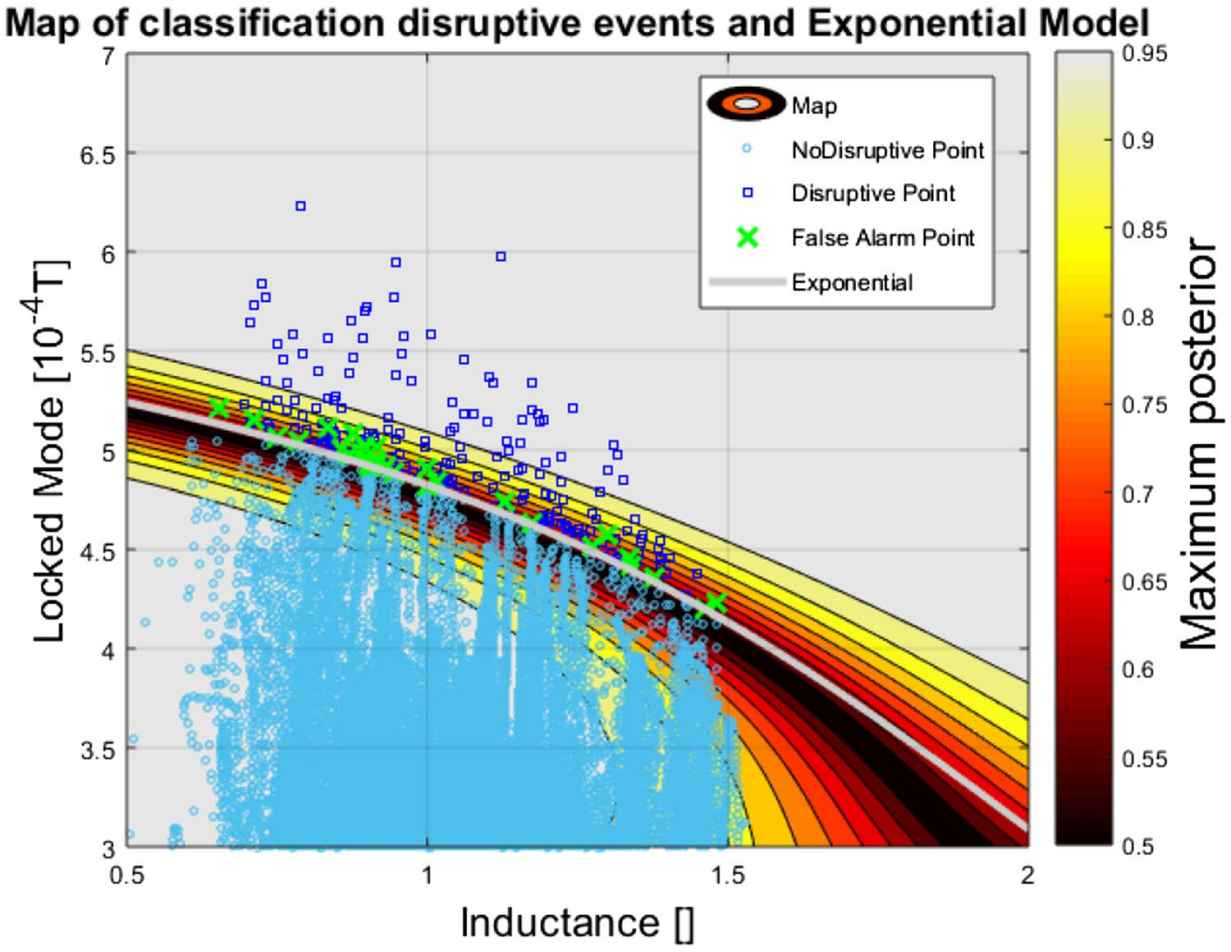
Plot of the safe and disruptive regions of the operational space in JET at the beginning of operation with the ILW. The colour code represents the posterior probability of the classifier. The light blue circles are all the non-disruptive shots (10 random time slices for each shot). The blue squares are the data of the disruptive shots at the time slice when the predictor triggers the alarm. The green crosses are the false alarms. The white line represents Eq. (7). Figure created using MATLAB R2019a, https://it.mathworks.com/products/matlab.html.

The just described procedure has been tested using a variety of ML tools, ranging from clustering to probabilistic classifiers. In detail, the machine learning technology, used to obtain the results presented in this section, is probabilistic Support Vector Machines (SVM). The database investigated comprises 187 disruptions and 1200 safe shots, belonging to campaigns C29-C30 at the beginning of JET operation with the new ITER Like Wall (ILW). The separation between the disruptive and safe regions of the operational space, in the plane of the locked mode amplitude and the internal inductance, is reported in Fig. 3. The following equation has been retained as a good compromise between complexity and accuracy:

7 $$ LM\left( {l_{i} } \right) = a_{0} \exp \left( {a_{1} l_{i}^{{a_{2} }} } \right) $$

where *LM* is the amplitude of the locked mode expressed in 10^–4^ T, *l*_*i*_ the internal inductance and the coefficients assume the values:

$$ \begin{aligned} a_{0} & = 5.4128 \pm 0.0031; \\ a_{1} & = - 0.11614 \pm 0.00085; \\ a_{2} & = 2.21 \pm 0.011; \\ \end{aligned} $$

The performance of the previous equation reproduces very well the one of the original SVM model as can be appreciated from Table 4, where the traditional indicators used to quantify the quality of predictors are reported. In terms of interpretability, Eq. (7) should be compared with an SVM model of tens of Gaussian functions centred on the support vectors. In this application, therefore, the results are particularly positive, because the dramatic increase in interpretability does not imply a loss of accuracy. From the point of view of the physics, the relation between the locked mode amplitude and the current profile, identified by SR via GP, is an important aspect, which has to be included in any theoretical model of disruption physics^41^.

**Table 4:** The figures of merit obtained using SVM and Eq. (7), for JET Campaigns C29–C30. Missed alarms are disruptions not detected or detected after the beginning of the current quench. Tardy alarms are disruptions detected less than 10 ms from the beginning of the current quench. False alarms are triggered during safe discharges. The last column reports the mean warning time.

Method	Success rate	Missed	Early	Tardy	False	Mean (ms)
SVM	97.9% (183/187)	0% (0/187)	0% (0/187)	2.1% (4/187)	2.8% (29/1020)	335
Equation (7)	97.9% (183/187)	0% (0/187)	0% (0/187)	2.1% (4/187)	2.8% (29/1020)	336

## Conclusions

The proposed methodology is meant to support theory formulation starting from the data, when the complexity of the phenomena to be studied is so severe that it is difficult or impossible to devise models from first principles. It is to be considered a complement to traditional hypothesis driven modelling. Indeed, the developed tools mathematize the derivation of theoretical models directly from the data, in analogy with the analogous process of formulating new models from existing ones in the hypothesis driven approach. Therefore SR via GP at least alleviates a traditional weakness of present day research, since so far deriving models from data has been more an art than a scientific procedure. The proposed techniques make systematic use of the most advanced machine learning tools, which are proving so successful in society at large. Application of Symbolic Regression and Genetic Programming has proved invaluable to obtain results of a physically meaningful and interpretable form. More advanced versions of the tools, with a more sophisticated treatment of the errors in the measurements, using the Geodesic Distance, are also available^45–47^. The proposed approach has also found various applications in several branches of physics, such as atmospheric physics and remote sensing^48,49^, and not only in the field of thermonuclear plasmas. In terms of future applications, it is planned to combine SR via GP also with neural networks of complex topology, to profit from the great exploratory power and flexibility of deep learning^50,51^. Upgrades of the methodology to address times series with memory, recursive functions^52–55^ and distributed quantities are also quite advanced.
